# Next-generation sequencing reveals a new mutation in the *LTBP2* gene associated with microspherophakia in a Spanish family

**DOI:** 10.1186/s12881-018-0590-0

**Published:** 2018-05-11

**Authors:** Laura Alías, Jaume Crespi, Lidia González-Quereda, Jesús Téllez, Elisabeth Martínez, Sara Bernal, Ma Pia Gallano

**Affiliations:** 1 Department Genetics, Hospital de la Santa Creu i Sant Pau, Pare Claret, 167, 08025 Barcelona, Spain; 2 CIBERER (U705), Barcelona, Spain; 3 Department Ophthalmology, Hospital de la Santa Creu i Sant Pau, Barcelona, Spain

**Keywords:** Microspherophakia, *LTBP2* gene, NGS, Clinical exome sequencing, TruSight one sequencing panel

## Abstract

**Background:**

Microspherophakia is a rare autosomal recessive eye disorder characterized by small spherical lens. It may present as an isolated finding or in association with other ocular and/or systemic disorders. This clinical and genetic heterogeneity requires the study of large genes (*ADAMTSL4, FBN1, LTBP2, ADAMTSL-10* and *ADAMTSL17*). The purpose of the present study is to identify the genetic cause of this pathology in a consanguineous Spanish family.

**Methods:**

A clinical exome sequencing experiment was executed by the *TruSight One*® *Sequencing Panel* (TSO) from Illumina©. Sanger sequencing was used to validate the NGS results.

**Results:**

Only the insertion of an adenine in exon 36 of the *LTBP2* gene (c.5439_5440insA) was associated with pathogenicity. This new mutation was validated by Sanger sequencing and segregation analysis was also performed. Haplotype analyses using the polymorphic markers *D*14*S*1025, *D14S43* and *D14S999* close to the *LTBP2* gene indicated identity by descent in this family.

**Conclusion:**

We describe the first case of a microspherophakia phenotype associated with a novel homozygous mutation in the *LTBP*2 gene in a consanguineous Caucasian family by means of NGS technology.

## Background

Microspherophakia (MSP, OMIM 251750) is a rare autosomal recessive (AR) eye disorder characterized by small spherical lens. It may present either as an isolated finding or in association with other ocular anomalies such as megalocornea, ectopia lentis and secondary glaucoma, or with hereditary systemic disorders such as Marfan syndrome and Weill-Marchesani syndrome. These two latter conditions are caused by mutations in the *FBN1* gene, whose protein product fibrillin-1 (FBN1) is a major structural component of the microfibrils. Three additional genes of the *ADAMTS* family (4, 10 and 17) have been associated with some of these conditions, from isolated ectopia lentis to Weill-Marchesani syndrome. In patients with microspherophakia or other ocular anomalies, such as megalocornea, myopia, congenital primary glaucoma or secondary glaucoma, mutations have been found in the *LTBP2* gene. Isolated microspherophakia (IM), without any other ocular feature, has been recently been linked to the *LTBP2* gene [1]. This latent transforming growth factor beta binding protein 2 (*LTBP2*) gene is considered one of the major known causative genes for MSP.

The *FBN1* gene, with 65 exons extending over 200 kb of genomic DNA, codes for the main protein of extra-cellular microfibrils: Fibrillin-1. It is an extracellular matrix glycoprotein that serves as a structural component of calcium-binding microfibrils. These microfibrils provide force-bearing structural support in connective tissue. The *LTBP2* gene, 114 kb long and organized into 22 exons, is the largest member of the *LTBP* family. The coded protein shows strong structural homologues with the fibrillins. Because the C-terminal region of the LTBP2 protein specifically binds to the N-terminal region of fibrillin-1, this protein may have a structural role in elastic-fiber architectural organization for LTBP2 [2]. The *ADAMTSL4* gene (22 exons/10 Kb of genomic DNA) [3], the *ADAMTS10* gene (26 exons/30 Kb of genomic DNA) [4] and the *ADAMTS17* gene (24 exons /370Kb) [5] belong to a family of extracellular matrix proteases. It has been postulated that these proteins play either a structural or a regulatory role in the microfibrillar network [6].

The present work describes for the first time the association of a mutation in the *LTBP2* gene (c.5439_5440insA) with an isolated microspherophakia phenotype in a consanguineous Caucasian family.

## Methods

### Clinical evaluation

**Fig. 1:**
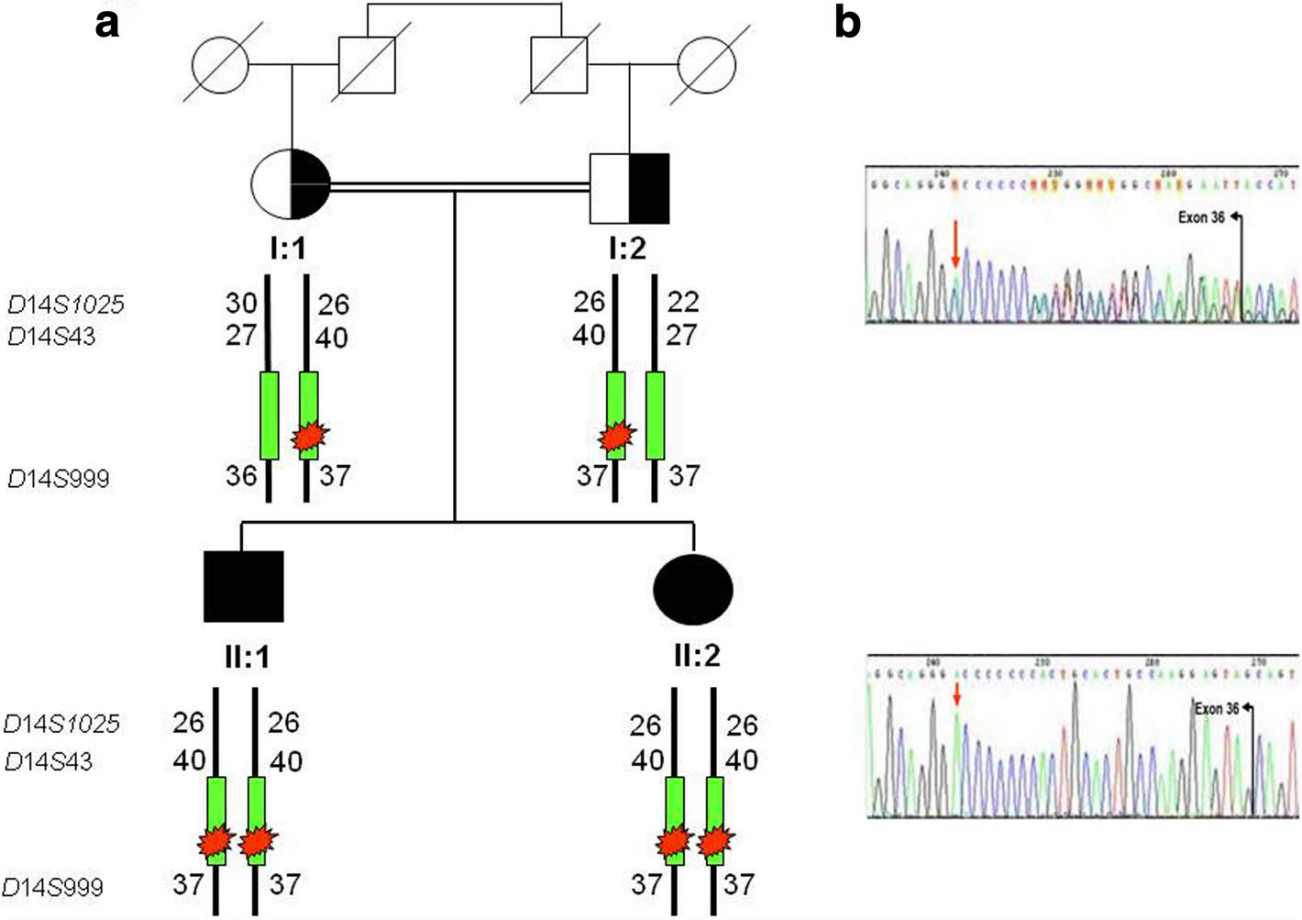
**a** Pedigree of the consanguineous Spanish family with microspherophakia and their haplotype study by *D*14*S*1025, *D*14*S*43 and *D*14*S*999 markers close to the *LTBP2* gene on chromosome 14. **b** Electropherograms obtained by Sanger sequencing of the *LTBP2* gene showing the c.5439_5440insA mutation detected. The sequences of the parents, where the mutation was detected in heterozygous state, are shown above, and the sequences of the affected patients, in which the added Adenine showed a homozygous peak, are shown below

We studied two siblings from a consanguineous Spanish family with microspherophakia (MSP) (Fig. 1). Both patients underwent a complete ophthalmic and systemic workup, including eco-cardiography and homocystinuria screening. Neither had any cardiovascular, metabolic or musculo skeletal abnormalities.

Bilateral slit lamp biomicroscopy, intraocular pressure (IOP) measurement with Goldmann applanation tonometer, gonioscopy and fundus examination (through a dilated pupil and using +90D Volk lens) were performed. In both siblings, the slit-lamp biomicroscopy revealed that lenses were smaller and more spherical than normal (MSP). The absence of enlarged cornea, buphthalmos, abnormal angle structures or increased IOP excluded primary congenital glaucoma. The diagnosis of isolated MSP was confirmed by ultrasound biomicroscopy (UBM, Sonomed Inc., New York, USA).

### Genetic studies

Genomic DNA was automatically extracted from peripheral leukocytes using the salting out procedure (Autopure, Qiagen). To screen for the presence of mutations in the *ADAMTSL4* gene, we sequenced all 20 exons and exon-intron boundaries of the gene (BigDye v1.1 Terminator Reaction Kit on an ABI Prism ® 3500 Dx Capillary DNA Sequencer unit according to manufacturer’s protocol, Life Technologies Corporation). The sequences of primers, annealing temperatures and PCR conditions were adapted from the literature [7].

To perform NGS analyses we used a clinical exome sequencing panel called *TruSight One*® *Sequencing Panel* (TSO) from Illumina©. This panel focuses on exonic regions harboring 4813 disease-causing variants. We included all genes that have been associated to date to isolated ectopia lentis (*ADAMTS4*, *FBN1*, *LTBP2*, *ADAMTS10*, *ADAMTS17*) in the clinical exome panel. Amplified samples following the TSO protocol were loaded onto the MIseq instrument according to the manufacturer’s instructions (Illumina©). VariantStudio software® (Illumina©) allowed us to analyze the files from the experiment. Sanger sequencing was used to validate the NGS results.

To investigate whether IBD (identity by descent) occurs in the studied family, we performed haplotype analysis of the region containing the *LTBP2* gene. We studied three polymorphic markers (*D*14*S*1025, *D*14*S*43 and *D*14*S*999) located on the 5′ up-stream region of the gene. The fragments amplified by dye-labelled primers of the *D*14*S*1025, *D*14*S*43 and *D*14*S*999 markers were analyzed on an ABI Prism ® 3500 Dx Capillary DNA Sequencer (Life Technologies Corporation©). Genotypes were determined using the GeneScan® software package (Perkin Elmer-Applied Biosystems©).

All mutations and genetic variants were numbered according to the first translated base of the sequenced genes (GenBank entry NT_006713) and variant sequences were designated according to standard nomenclature guidelines [8].

### In silico studies

To investigate the functional impact of the genomic variations found in the present study, we used the ALAMUT® VISUAL software. This software uses the following relevant prediction tools: i) Splice Site Finder-like, MaxEntScan, NNSPLICE, GeneSplicer, Human Splicing Finder and ESE for splicing prediction ii) Align GVGD, SIFT, MutationTaster, PolyPhen-2 and KD4v for missense prediction.

To check all the variants detected in this study the ExAc, the 1000 Genomes Project and the CSVS (Collaborative Spanish Variant Server) databases were consulted.

## Results

### Clinical results

**Fig. 2:**
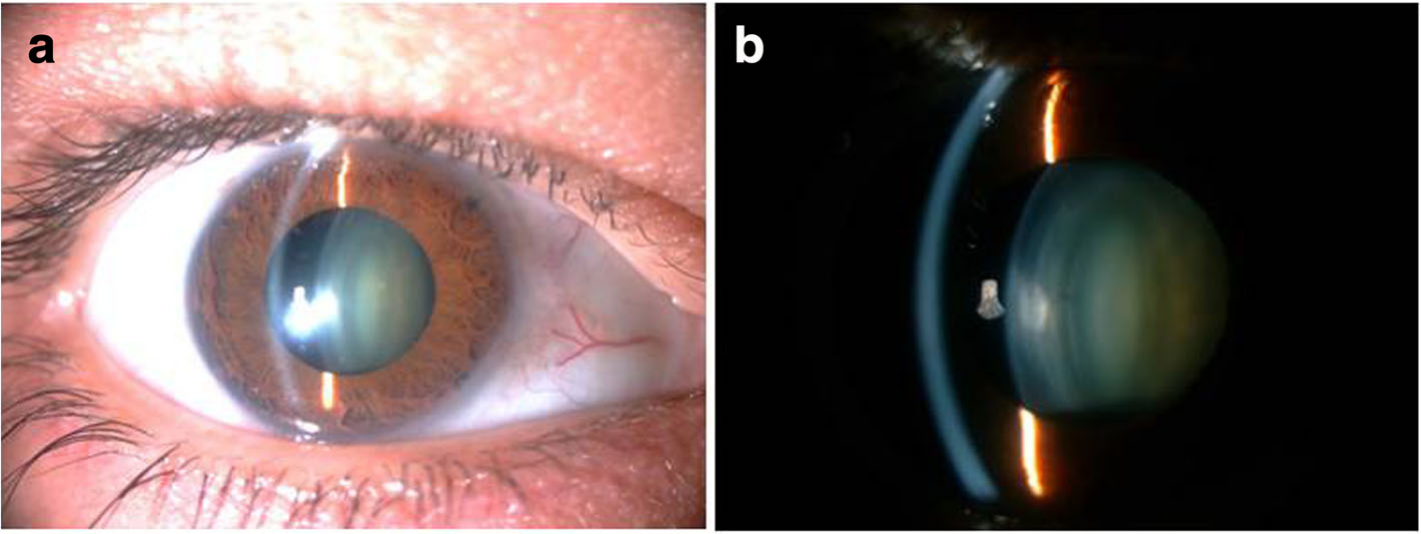
**a** Slit lamp photography. **b** A left eye section performed in patient II.1. shows a small nuclear cataract. The lens is small in diameter and spherical in shape

**Fig. 3:**
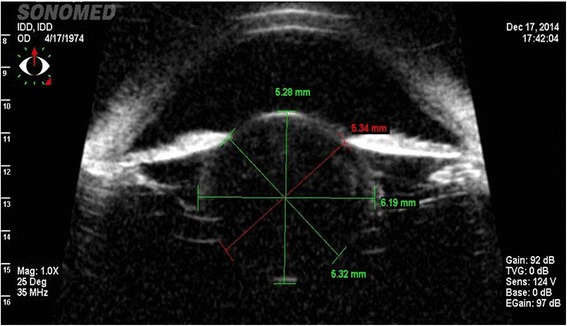
Ultrasound biomicroscopy performed in patient II:1. The analysis showed a thickness of 5,32 mm (OD) and 5,35 mm (OS), respectively, and evidence of missing and stretched zonular fibres

Figure 1 shows the pedigree of the family studied. Patient II:1 was a 42-year-old man who was referred to our department for progressive visual loss and high lenticular myopia. His medical history was unremarkable. Visual acuity was 20/50 in both eyes. Refractive error was − 22.00/− 0.75 × 78^0^ (OD) and − 21.00/ -0,50 × 100^0^ (OS). Intraocular pressure was 19 mmHg and 20 mmHg in OD and OS, respectively. Gonioscopy examination showed open angle grade IV 360°. Ophthalmic examination findings were compatible with MSP. The lens had a central nuclear cataract with notable phacodonesis but without subluxation in either eye (Fig. 2). Ultrasound biomicroscopy showed that the equatorial lens diameter was small with an increased antero-posterior diameter, consistent with microspherophakia. UBM also detected the presence of missing and stretched zonular fibers which were compatible with clinical phacodonesis (Fig. 3). A fundoscopic exam showed that optic disk appearance was normal and no pathology was observed. The patient underwent bilateral lensectomy with posterior iris claw intraocular lens implantation that restored vision to 20/20 in both eyes.

Patient II:2 was an asymptomatic 39-year-old woman and the proband’s younger sister. Her visual acuity was normal and IOP was 18 mmHg in both eyes. Gonioscopy examination showed open angle grade IV bilaterally. The ophthalmic examination showed microspherophakia without cataracts or lens dislocation. The fundoscopic exam of the posterior segment was unremarkable and the cup-disk ratio was within normal limits in both eyes.

### Genetic results

Taking into account that the *ADAMTSL4* gene is considered responsible for most cases of isolated ectopia lentis in the European population [9], we started the present study by sequencing all 20 exons and exon-intron boundaries of this gene. We identified several variants from the reference sequence (Table 1), but none met the requirements to be considered as the molecular cause of the disease in the consanguineous family studied.

**Table 1:** *ADAMTSL4* genetic variants detected in the IEL patients by Sanger sequencing of this gene

	Exon/Intron	DNA variant	cDNA	Protein	SNP	MAF	State
Patient 1	exon 6	g.150526044 G > C	c.577G > C	p.Ala193Pro	rs41317515	0,443	Het
	intron 8	g.150527292 G > A	c.1303 + 182G > A		rs9659061	0,452	Het
	intron 8	g.150527294 C > T	c.1303 + 184C > T		rs12124948	0,338	Het
	intron 8	g.150527703_150527704 ins TCAT	c.1304-202_1304-201insTCAT				Het
	intron 11	g.150529323_150529324 ins TT	c.1818 + 54_1818 + 55insTT				Het
	exon 16	g.150558532 T > C	c.2511 T > C	p.Asn837Asn	rs1088382	0,156	Hom
	exon 16	g.150558574 G > A	c.2553G > A	p.Pro851Pro	rs10749657	0,118	Het
	intron 16	g.150558694 C > T	c.2628 + 45C > T		rs10749658	0,065	Hom
Patient 2	exon 6	g.150526044 G > C	c.577G > C	p.Ala193Pro	rs41317515	0,443	Het
	intron 8	g.150527292 G > A	c.1303 + 182G > A		rs9659061	0,452	Het
	intron 8	g.150527294 C > T	c.1303 + 184C > T		rs12124948	0,338	Het
	intron 8	g.150527703_150527704insTCAT	c.1304-202_1304-201insTCAT				Het
	intron 11	g.150529323_150529324insTT	c.1818 + 54_1818 + 55insTT				Het
	exon 16	g.150558532 T > C	c.2511 T > C	p.Asn837Asn	rs1088382	0,156	Hom
	exon 16	g.150558574 G > A	c.2553G > A	p.Pro851Pro	rs10749657	0,118	Het
	intron 16	g.150558694 C > T	c.2628 + 45C > T		rs10749658	0,065	Hom
	intron 16	g.150531380 T > C	c.2629-58 T > C		rs11204664	0,452	Het

*Het* Heterozygous state, *Hom* Homozygous state

All the genes associated with ectopia lentis phenotypes to date, (*ADAMTSL4*, *FBN1*, *LTBP2*, *ADAMTS10*, *ADAMTS17*) included in the clinical exome employed, revealed several genetic variants in these genes. We analyzed their sequences using VariantStudio software. Two different filters were applied to the NGS panel: a) genetic variants detected in the genes described in the literature causing isolated ectopia lentis; and b) genetic variants detected in any of the 4813 genes analyzed by the panel causing a frameshift or a nonsense mutation. Table 2 shows the genetic variants identified. Only one of them has been associated with a pathogenic effect; the insertion of an adenine in the g.74967613 position of the chromosome 14 (NM_000428: c.5439_5440insA) would cause the appearance of a premature stop codon 30 aminoacids later (p.Pro1814ThrfsX30). This frameshift mutation (c.5439_5440insA) in the *LTBP2* gene has not been previously reported.

**Table 2:**
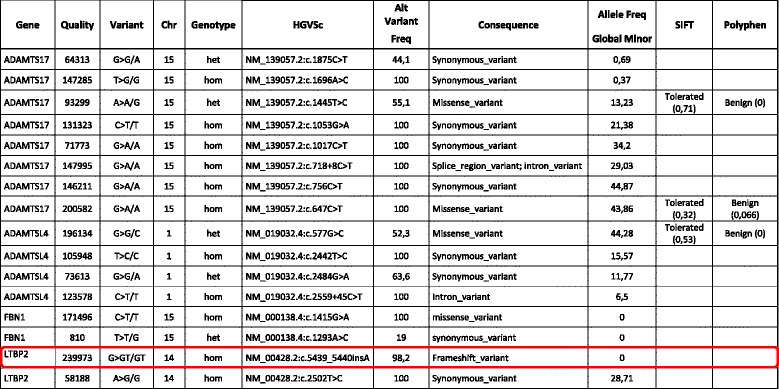
Results of the analyses of the TruSight One Panel using VariantStudio software

Genetic variants detected in the genes described in the literature as responsible for the IEL disease (*ADAMTSL4*, *FBN1*, *LTBP2*, *ADAMTS10*, *ADAMTS17*) with a missense, a frameshift or a nonsense mutation. The frameshift c.5439_5440insA mutation in exon 36 of the *LTBP2* gene, reported for the first time in this work, is indicated in red. In the “Genotype” column: Hom should read homozygous and Het should read heterozygous

The presence of the c.5439_5440insA mutation in exon 36 of the *LTBP2* gene was identified in all four family members by Sanger sequencing. As expected, the two patients were homozygous for the mutation while their parents showed this mutation in a heterozygous state. The study of the polymorphic markers (*D*14*S*1025, *D14S43* and *D14S999)* located next to the *LTBP2* gene demonstrated that identity by descent (IBD) occurred in this family (Fig. 1).

Finally, we ruled out the presence of the c.5439_5440insA mutation in DNA samples of a Spanish control population (*n* = 100 blood donors) by Sanger Sequencing. Furthermore, c.5439_5440insA had not been previously reported in the ExAc, the 1000 Genomes Project or the CSVS databases.

## Discussion

The present work reports the clinical and genetic study of a Spanish consanguineous family with two cases of isolated microspherophakia. We identified the presence of a novel mutation in the *LTBP2* gene: c.5439_5440insA in the two affected siblings. A mutation in this gene causing isolated microspherophakia in Caucasian patients has not been reported previously.

The latest technological advances allow us to obtain NGS results of thousands of unrelated individuals. There are several databases that include the results of many different projects. All this information has allowed us to obtain an amazing statistical power, especially in the research on rare diseases. We have checked that the c.5439_5440insA mutation has not been reported in any of the databases consulted (ExAc, 1000 Genomes Project or CSVS). Thus, the presence of this mutation was not only excluded in the 100 control individuals sequenced during this study, but we were also able to exclude it in more than 67,000 unrelated individuals from the ExAc database (which includes the results of the 1000 genomes project) and in more than 1582 unrelated Spanish individuals from CSVS.

To date, mutations in the *LTBP2* gene have been associated with several types of glaucoma. In primary congenital glaucoma (PCG), null mutations in LTBP2 were reported in four consanguineous families of Gypsy ethnicity from Pakistan [2], in three unrelated Iranian families [10], and, more recently, two novel mutations in consanguineous families of Pakistani ancestry were identified using WES [11]. In primary open angle glaucoma, five putative disease-contributing or risk factor mutations in *LTBP2* were observed in 42 Iranian patients [12]. All these findings will contribute to the understanding of the genotype-phenotype correlation in patients with *LBPT2* mutations.

Recently, in three consanguineous Saudi families with congenital megalocornea with zonular weakness and childhood lens-related secondary glaucoma it was found that ocular anomaly segregated with homozygous *LTBP2* mutations [13]. In two families of Moroccan and Macedonian descent, biallelic null *LTBP2* mutations were identified in patients with megalocornea, spherophakia, and secondary glaucoma [14].

Involvement of *LTBP2* mutations in hereditary systemic disorders, such as Marfan or Weill-Marchesani syndromes, has also been studied. In a meeting abstract, Mathews et al. reported a missense mutation in the *LTBP2* gene in a patient with an atypical MFS. More recently, in their study of Iranian patients, Haji-Seyed-Javadi et al. showed that *LTBP2* is a causative gene for Weill-Marchesani syndrome and suggested it plays a role in some clinical features observed in Marfan syndrome patients [15].

**Fig. 4:**
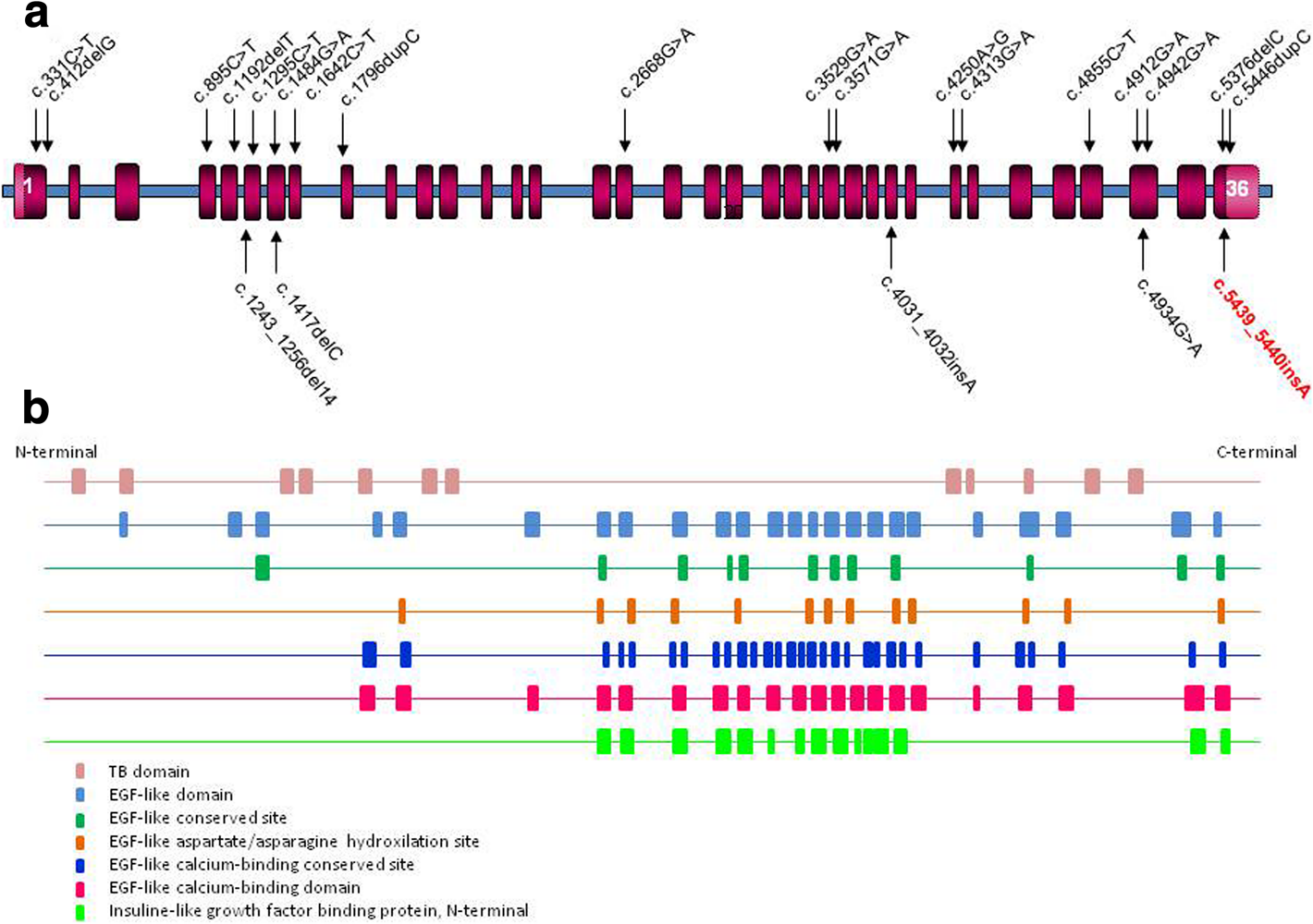
**a** Schematic representation of the mutations reported along the *LTBP2* gene. The novel c.5439_5440insA mutation in exon 36 at the end of this gene is shown in red. **b** Schematic representation of the functional domains in the LTBP2 protein

**Fig. 5:**
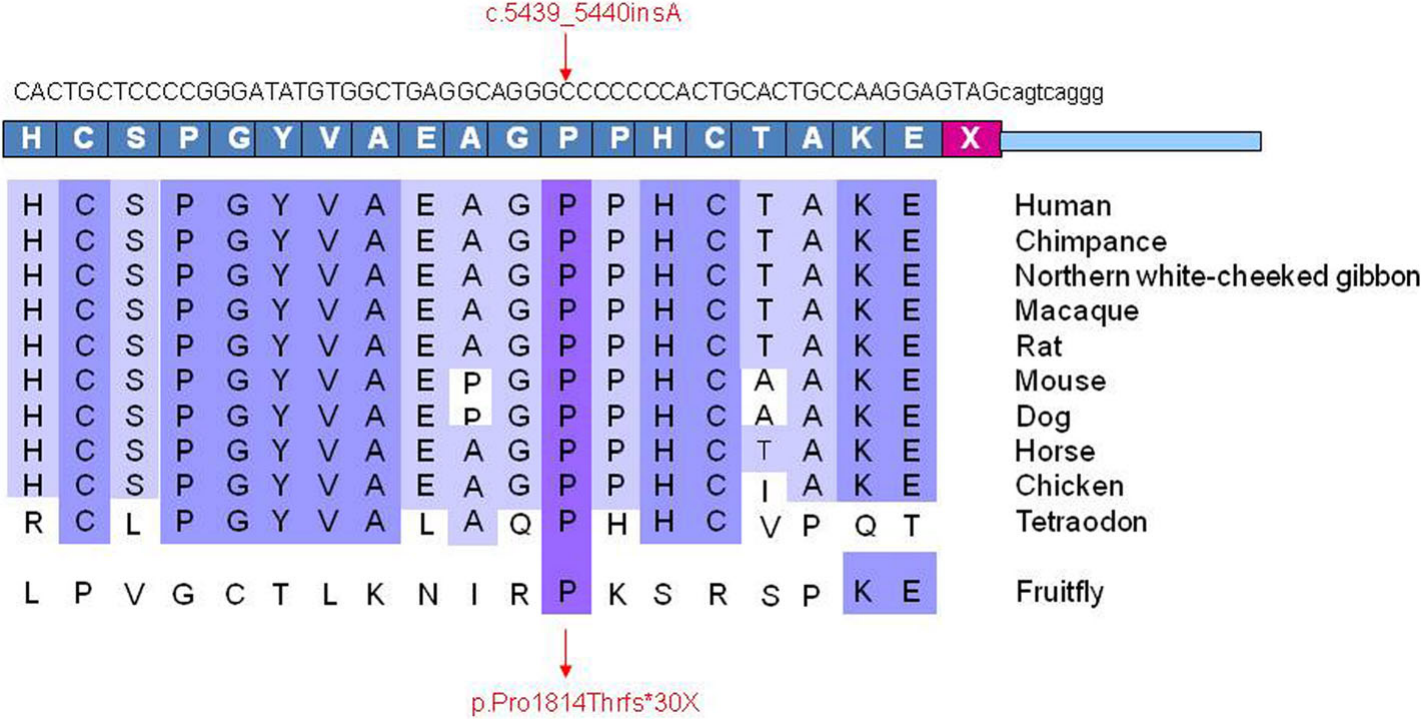
Conservation analysis across the species of a fragment from exon 36 of the *LTBP2* gene where the c.5439_5440insA mutation is located. The genetic region that encompasses the altered amino acid remains highly conserved between species

Figure 4 schematizes the location of all the *LTBP2* mutations published. The correlation between the localization of the mutations and their clinical manifestations has not yet been clearly established. However, it is reasonable to speculate with the intervention of regulatory sequences or other genes with a phenotype-modifying effect, especially in those syndromic phenotype diseases. Nonetheless, the two mutations associated only with microspherophakia, c.5446dupC and c.5439_5440insA are located in the last exon of the *LTBP2* gene. In silico studies predict that both frameshift mutations would elongate their respective mutant proteins, producing changes in the amino acid composition of the C-terminal domain of the native protein. Because the interaction between the LTBP2 and the Fibrillin-1 proteins is close to this LTBP2 C-terminal region, we could speculate that the instability of the zonular fibers characteristic in microspherophakia is due to the loss of the association between these two proteins. It has been postulated that the LTBP2 interaction with some members of the TGF-beta family could regulate microfibril storage in the extracellular matrix. An altered regulation due to the presence of an abnormal LTBP2 protein might thus prevent normal growth of the lenses, characteristic of the microspherophakia phenotype [12]. Furthermore, the alignment of the sequences from different species depicted in Fig. 5 shows that the c.5439_5440insA mutation is located in highly conserved residues, indicating its importance for the functionality of the LTBP2 protein.

All previously known cases with mutations in the *LTBP*2 gene were of Arab ethnicity. The present work describes an isolated microspherophakia phenotype due to a new homozygous mutation in the *LTBP*2 gene in a Spanish consanguineous family. These new data highlight the value of investigating the involvement of the *LTBP2* gene in cases with an isolated microspherophakia phenotype.

The present work reinforces the fact that the new technologies of exome sequencing are becoming a common molecular diagnostic test for rare genetic disorders. Indeed, clinical exome sequencing proved to be an effective tool to identify the causative mutation in the family studied.

## Conclusions

We describe a consanguineous Caucasian family showing a microspherophakia phenotype. Next-generation sequencing detected a homozygous novel frameshift mutation in the *LTBP2* gene present in the two affected patients. This is the first report of the association of a mutation in the *LTBP2* gene and isolated microspherophakia in Caucasians.

## Abbreviations

AR: Autosomal recessive
CSVS: Collaborative Spanish Variant Server
FBN1: Fibrillin-1
IBD: Identity by descent
IM: Isolated microspherophakia
IOP: Intraocular pressure measurement
MFS: Marfan syndrome
MSP: Microspherophakia
NGS: Next-generation sequencing
OD: Oculus dexter (the right eye)
OS: Oculus sinister (the left eye)
PCG: Primary congenital glaucoma
TSO: *TruSight One*® *Sequencing Panel*
UBM: Ultrasound biomicroscopy

## References

[1] Kumar A, Duvvari MR, Prabhakaran VC, Shetty JS, Murthy GJ, Blanton SH (2010). A homozygous mutation in LTBP2 causes isolated microspherophakia. Hum Genet..

[2] Ali M, McKibbin M, Booth A, Parry DA, Jain P, Riazuddin SA (2009). Null mutations in LTBP2 cause primary congenital glaucoma. Am J Hum Genet.

[3] Greene VB, Stoetzel C, Pelletier V, Perdomo-Trujillo Y, Liebermann L, Marion V (2010). Confirmation of ADAMTSL4 mutations for autosomal recessive isolated bilateral ectopia lentis. Ophthalmic Genet.

[4] Dagoneau N, Benoist-Lasselin C, Huber C, Faivre L, Megarbane A, Alswaid A (2004). ADAMTS10 mutations in autosomal recessive Weill-Marchesani syndrome. Am J Hum Genet.

[5] Le Goff C, Cormier-Daire V (2011). The ADAMTS (L) family and human genetic disorders. Hum Mol Genet.

[6] Buchner DA, Meisler MH (2003). TSRC1, a widely expressed gene containing seven thrombospondin type I repeats. Gene.

[7] Ahram D, Sato TS, Kohilan A, Tayeh M, Chen S, Leal S (2009). A homozygous mutation in ADAMTSL4 causes autosomal-recessive isolated ectopia lentis. Am J Hum Genet.

[8] den Dunnen JT, Dalgleish R, Maglott DR, Hart RK, Greenblatt MS, McGowan-Jordan J (2016). HGVS recommendations for the description of sequence variants: 2016 update. Hum Mutat.

[9] Neuhann TM, Artelt J, Neuhann TF, Tinschert S, Rump A (2011). A homozygous microdeletion within ADAMTSL4 in patients with isolated ectopia lentis: evidence of a founder mutation. Invest Ophthalmol Vis Sci.

[10] Narooie-Nejad M, Paylakhi SH, Shojaee S, Fazlali Z, Rezaei Kanavi M, Nilforushan N (2009). Loss of function mutations in the gene encoding latent transforming growth factor beta binding protein 2, LTBP2, cause primary congenital glaucoma. Hum Mol Genet.

[11] Micheal S, Siddiqui SN, Zafar SN, Iqbal A, Khan MI, den Hollander AI (2016). Identification of novel variants in LTBP2 and PXDN using whole-exome sequencing in developmental and congenital Glaucoma. PLoS One.

[12] Jelodari-Mamaghani S, Haji-Seyed-Javadi R, Suri F, Nilforushan N, Yazdani S, Kamyab K (2013). Contribution of the latent transforming growth factor-beta binding protein 2 gene to etiology of primary open angle glaucoma and pseudoexfoliation syndrome. Mol Vis.

[13] Khan AO, Aldahmesh MA, Alkuraya FS (2011). Congenital megalocornea with zonular weakness and childhood lens-related secondary glaucoma - a distinct phenotype caused by recessive LTBP2 mutations. Mol Vis.

[14] Desir J, Sznajer Y, Depasse F, Roulez F, Schrooyen M, Meire F (2010). LTBP2 null mutations in an autosomal recessive ocular syndrome with megalocornea, spherophakia, and secondary glaucoma. Eur J Hum Genet.

[15] Haji-Seyed-Javadi R, Jelodari-Mamaghani S, Paylakhi SH, Yazdani S, Nilforushan N, Fan JB (2012). LTBP2 mutations cause Weill-Marchesani and Weill-Marchesani-like syndrome and affect disruptions in the extracellular matrix. Hum Mutat.

